# Effect of Telehealth System on Glycemic Control in Children and Adolescents with Type 1 Diabetes

**DOI:** 10.4274/jcrpe.galenos.2018.2018.0017

**Published:** 2019-02-20

**Authors:** Esra Döğer, Rukiye Bozbulut, A. Şebnem Soysal Acar, Şebnem Ercan, Aylin Kılınç Uğurlu, Emine Demet Akbaş, Aysun Bideci, Orhun Çamurdan, Peyami Cinaz

**Affiliations:** 1 Gazi University Faculty of Medicine, Department of Pediatric Endocrinology, Ankara, Turkey

**Keywords:** Type 1 diabetes, telehealth, diabetes team, HbA1c

## Abstract

**Objective:**

A close diabetes team-patient relationship is required for establishing satisfactory metabolic control. The purpose of this study was to investigate the effect of a telehealth system on diabetes control.

**Methods:**

The study was carried out between June 2015 and January 2016 at the Gazi University Faculty of Medicine, Pediatric Endocrinology Department. The telehealth system was developed by the diabetes team. The demographic characteristics, frequency of use and hemoglobin A1c (HbA1c) changes of type 1 diabetic (T1DM) patients using this communication network were analysed.

**Results:**

Eighty two patients [43 (52.4%) females, mean (±standard deviation) age 10.89±4 years] used the telehealth system. Fourteen (17.1%) of the cases were on pump therapy and 59 (72.0%) were counting carbohydrates. The individuals with diabetes or their families preferred WhatsApp communication. Whatsapp provided a means for instant messaging in most instances (57.3%), contact with diabetes education nurse (32.9%) and consultation with the diabetes team about insulin doses and blood glucose regulation (42.7%). HbA1c values after six months were significantly lower in patients/parents calling frequently (p<0.001) compared with HbA1c values recorded at the beginning of the study.

**Conclusion:**

Increase in frequency of counselling by the diabetes team led to improved blood glucose control in T1DM patients. A telehealth system is useful for early detection of the need for changes in treatment and for intervention. It also promoted better self care.

**What is already known on this topic?**

In the management of diabetic patients, diabetes education and communication with the diabetes team is as important as medical treatment. Telehealth systems facilitate communication with the diabetes team.

**What this study adds?**

The use of telehealth systems in Turkish children and adolescents with type 1 diabetes mellitus was shown to improve glycemic control.

## Introduction

Diabetes management is clinically demanding (1). Insulin treatment, healthy eating appropriate for the diabetic state and physical activity are important in diabetes management. Diabetic patients need to learn how to keep these factors in balance (2). A close health professional-patient relationship, individualized care and education are essential for attaining good glycemic control in diabetic patients (3). Educational interventions in children and adolescents with diabetes are known to enhance glycemic control and psychosocial health (4).

Diabetes education should involve both the diabetic child and his/her family and should be provided by a team of professionals including the physician, nurse, dietitian and psychologist (5). To maintain their blood glucose at normal levels and thus prevent complications of diabetes, these patients should be able to contact the diabetes team frequently and easily and be able to access health services at all times (2,3). However, reasons such as failure to increase the number of health professionals in line with the increase in the number of patients with diabetes has led to decreased quality of service for providing a full care package, including the educational interventions necessary for the self-management of patients (6,7). Inadequacies in the training of the medical team may be another factor contributing to poorer levels of service offered in diabetic care (8). Moreover, diabetic children residing in rural areas are faced with greater difficulty in accessing healthcare (2,6). Providing health information using newer communication technologies will help to overcome some of the difficulties cited above. This type of rapid communication provides greater flexibility in time and means for contacting the healthcare team, thus decreasing the number of patients presenting to outpatients. It will also lead to a decrease in the patient education tasks required from physicians and eventually to a decrease in the need for hospital care and lower financial costs (9,10,11,12).

The use of information technology and providing online education are recommended in undergraduate and postgraduate educational programs for all health workers, including continuing education programs (2). Ever more widespread use of the internet enables its greater use in the field of education (13). Web-based education should also be provided because it provides an accessible and permanent record of information for patients (14). Web-based applications also enable fast and effective communication with health professionals. Internet communication programs also serve rural patients, allowing them to contact health professionals from their home environment, thus decreasing the time and expenses spent in transportation (6,15,16). Another positive feature of these programs is that the patient can receive feedback and suggestions in line with his/her requirements very rapidly. Moreover, it has been reported that communications between the diabetes team and diabetic patients by phone and/or text messaging (SMS) using cellular phones increased compliance to treatment and enhanced glucose control (17,18,19).

The purpose of this study was to assess the effects of counselling services offered to diabetics by the diabetes team via communication networks (internet, phone) on diabetes control.

## Methods

The study was conducted between June 2015 and January 2016 at the Gazi University Faculty of Medicine Pediatric Endocrinology Department, Ankara. The telehealth system was developed in-house by the diabetes team. The demographic characteristics, frequency of use and hemoglobin A1c (HbA1c) changes over six months of use using this communication network were recorded for type 1 diabetic (T1DM) patients.

The telehealth system used in the study was developed by a pediatric diabetes team which included a nurse, dietician, psychologist and physician. Counselling was conducted via communication networks including the internet and smart phones. Counselling hours were scheduled for 11:30-14:30 and 21:00-24:00 hours. Oral communications were recorded for later analysis.

The purpose of the study was explained to each participant and written informed consent was obtained. The study procedures were in accordance with the Declaration of Helsinki. Prior to the study, families consented to statistical analysis and publishing of the data with the exception of any confidential communications. The study protocol was approved by Gazi University Ethics Committee (with the approval number: 583.10.09.2018).

The analyses were based on variables such as call frequency, duration of the diabetes, use of infusion pump or carbohydrate counting. We obtained the mean from frequency distribution of the patients who reached us and we grouped them according to it. Patients/parents calling daily, 5-6 times a week, 1-2 weekly or once every 15 days were identified as the frequent caller group, while those calling once a month, every two months or once every three months were identified as the infrequent caller group. 

Demographic characteristics and patient history information were collected from patient files. HbA1c levels at baseline and at follow-up were evaluated.

### Statistical Analysis

Statistical analyses were carried out by using the Statistical Package for Social Sciences (SPSS version 21.0, IBM Inc., Chicago, Ill., USA). The conformity to normal distribution of numerical data (age and HbA1c values) was tested with the Shapiro-Wilk test.

In the comparison of the numerical data of dependent paired groups (HbA1c values changes after six months for both frequent and rare call groups), the paired t-test was used for data with normal distribution.

Patients were grouped according to HbA1c levels after six months (<7.5%, 5-9% and >9% group), rare and frequent call groups on HbA1c levels after six months.

To examine the effects on HbA1c levels after six months of the call frequency groups, cross-reference tables were formed and evaluation was made with the chi-square test.

For categorical data such as demographic and communication features, tables were formed and values were presented as number of cases and percentage. For all the statistical calculations a value of p<0.05 was accepted as statistically significant.

## Results

The study was conducted on diabetic children aged between two and 18 years (mean ± standard deviation=10.89±4.00) consisting of 43 girls (52.4%) and 39 boys (47.6%) of comparable ages. 

Eleven (13.4%) of the participants were newly diagnosed diabetics (<1 year), 36 (43.9%) were patients with diabetes for 1-3 years, 19 (32.3%) for 4-6 years, and 16 (19.5%) had been diagnosed ≥7 years earlier. In the study group, 14 (17.1%) were using pumps while 59 (72.0%) counted carbohydrates. Of the participants 78 (95.1%) attended their check-up visits regularly and 11 (13.4%) had suffered from episodes of ketoacidosis after diagnosis. 

Parental data are given in Table 1.

In Table 2, the communication network used by the children or by other family members contacting the diabetes team, frequency of counselling, the most consulted diabetes team members and the purpose of the call are shown. 

There was a preference for using WhatsApp (57.3%) which is an instant messaging service, to contact the diabetes education nurse (32.9%) or consult with the diabetes team most frequently (42.7%) on their insulin dose and glucose regulation. Frequency of use of WhatsApp (28.0%) was higher than the other means of communication. 

In Table 3, HbA1c levels were compared between the call frequency groups (frequent versus infrequent callers, defined above). It was found that HbA1c values at baseline compared with after six months were lower in patients calling frequently (p<0.001).

Table 4 shows that HbA1c levels of the diabetic patients consulting with the diabetes team by using the telehealth method decreased significantly after six months. It was also observed that 32/36 (89%) of individuals with diabetes whose HbA1c value was lower than 7.5% consulted frequently while only 6/26 (23%) of patients whose HbA1c value was >9% consulted frequently.

## Discussion

The use of communication technologies as a means of providing healthcare services has increased the ability of patients living far from health centres to obtain information and advice and removed many of the barriers for other patients trying to access healthcare services (20). The telehealth system enables the use of information storage and delivery in the form of audio, image, speech or video through a range of communication technologies such as landline, satellite connection and cellular phone. This system provides duplex audiovisual communication between health professionals and patient (21).

In this study, communication between pediatric T1DM patients, their families and the diabetes therapy team via WhatsApp, phone and SMS was investigated. The results indicate that the diabetes team was contacted most frequently via WhatsApp, an intermessaging and file transfer/sending program which uses the internet (57.3%) and phone (23.2%) (Table 2). Whatsapp is used in numerous fields including in the field of education. “We conjecture that the reason why the children and their families prefer WhatsApp is that they can easily communicate with the diabetes team via SMS, a voice mail system and audio and video calls using wifi connectivity. An additional and important benefit is that they can immediately share photos showing their blood glucose values. It was found that most frequently (64.6%) it is the mothers of diabetic children who communicate with the diabetes team. The second most frequent contact was directly from the diabetic patient (Table 2).

An American Diabetes Association (2014) report, emphasizes that the acute and chronic complications of diabetes can be prevented or delayed in diabetics who receive education on self-management and who are being supported regularly (22). Diabetes-oriented education increases the quality of life and glycemic control is also improved to an optimal level in T1DM children and adolescents (23). However, it is also important that this education be continuous and that it is reinforced at frequent intervals to be effective in terms of glycemic control (24). 

In recent years, the telehealth system was introduced for use in the treatment of chronic diseases such as diabetes. This system is both flexible and cost effective. The patients can benefit from one-to-one education sessions, offering social support and information on self-management (25). In this study, a decline in HbA1c values was observed as the frequency of consulting sessions with the diabetes team increased. While the initial mean HbA1c value of frequent callers was 8.30±1.16%, their mean HbA1c decreased to 7.45±0.87%. In contrast, while the initial HbA1c mean value of the rare callers group was 9.10±1.26%, after six months their mean HbA1c levels increased to 9.28±1.25%. In the study carried out by Thompson et al (17), T1DM were contacted by phone for 15 minutes over the course of three weeks and additionally when necessary and insulin dose adjustments were made by the diabetes nurse. They reported significant reduction in HbA1c values of the patients at the end of six months. Our results are consistent with this. Xu et al (26) reported that the telehealth system they applied to individuals with T1DM reduced the mean HbA1c and glucose variability, and that the specific diabetes care provided by this method was associated with time and cost savings and compliance with diabetes treatment. In a study reported by Kwon et al (3), in which diabetic patients were able to contact the diabetes team using SMS and the internet, a significant decrease in HbA1c values was reported after three months. Likewise, in the study carried out by Bin-Abbas et al (19) daily information messages regarding the procedures related to diabetes care, weekly interactive messages and optional multimedia messages were sent to diabetic patients. Significant decreases in postprandial glucose and HbA1c levels, in the frequency of hypoglycemic episodes and in blood glucose levels were observed.

It has been reported that web-based telehealth services improve patient satisfaction, patient compliance to diabetes treatment and clinical results (19,27). Telehealth systems which include individualized evaluations, supervision and skills development by feedback are reported to be more effective in improvement of glycemic control. In this present study, it was found that the most frequently consulted therapy team member was the diabetes nurse and the most frequently issue consulted about was insulin dose and blood glucose regulation (42.7%). Questions concerning carbohydrate counting (29.3%) and appropriate actions to be taken in the case of hyperglycemia and hypoglycemia (17.1%) were next most frequent. Questions about methods of insulin injection, technical problems with instruments, such as pump failure, message failure, battery running out, set change or blockage in set, adjustments in insulin dose on special days, for example birthdays, weddings or picnics and in special cases such as intercurrent illness were the least often consulted topics (10.1%; see Table 2). We hypothesise that the diabetes team making suggestions, providing information and giving online support to the patients over six months provided greater continuity of communication with the patients than previously and led to improved self-esteem. It was also noted that this continuity in communication ensured that the changes in the patient’s status were detected earlier than was previously happening and appropriate interventions were more rapid. It was observed that 88.9% of 36 individuals with diabetes whose HbA1c value after six months was below 7.5% had called frequently while 76.9% of those whose HbA1c value was above 9.5% had called rarely.

### Study Limitations

The short duration of the study could be construed as a limitation although significant differences were detected and have been detected in shorter duration studies than this one (3). The limited number of cases constitute a definite limitation of the study. A final limitation was that the our telehealth system did not belong to our institution and was not accessible 24 hours a day.

## Conclusion

Increased frequency of consulting with the diabetes team improved the control of blood glucose levels of T1DM patients. Guidelines published in recent years emphasize individualized treatment and a multidisciplinary approach in diabetes management. A telehealth system developed by the diabetes team was a useful system in early detection of problems and facilitated rapid intervention, thus enhancing the self-care. 

In general we suggest that there is a need to establish an institutional system to provide a regular, effective tele-education service. The system should allow the whole team to access and monitor the patient’s entries simultaneously for a synchronized intervention.

## Figures and Tables

**Table 1:**
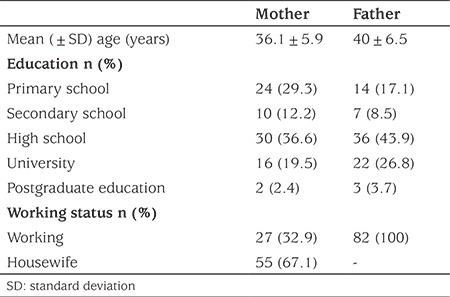
Demographic data of the parents

**Table 2:**
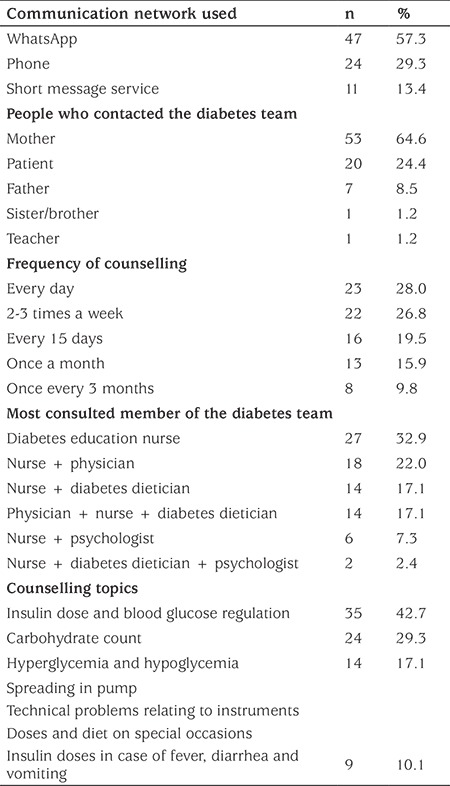
Communication features

**Table 3:**
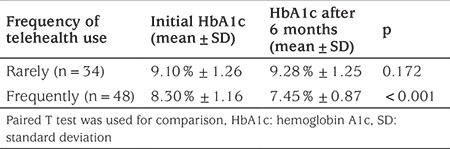
Hemoglobin A1c values by frequency of calling

**Table 4:**
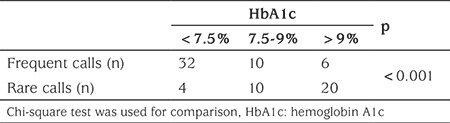
Comparison of call frequency groups (rare vs. frequent) on hemoglobin A1c levels after six months

## References

[1] Nylander C, Tindberg Y, Haas J, Swenne I, Torbjörnsdotter T, Akesson K, Örtqvist E, Gustafsson J, Fernell E (2018). Self‐and parent‐reported executive problems in adolescents with type 1 diabetes are associated with poor metabolic control and low physical activity. Pediatr Diabetes.

[2] Ayatollahi H, Hasannezhad M, Fard HS, Haghighi MK (2016). Type 1 diabetes self-management: developing a web-based telemedicine application. Health Inf Manag.

[3] Kwon HS, Cho JH, Kim HS, Lee JH, Song BR, Oh JA, Han JH, Kim HS, Cha BY, Lee KW, Son HY, Kang SK, Lee WC, Yoon KH (2004). Development of web-based diabetic patient management system using short message service (SMS). Diabetes Res Clin Pract.

[4] Lange K, Swift P, Pankowska E, Danne T (2014). Diabetes education in children and adolescents. Pediatr Diabetes.

[5] Silverstein J, Klingensmith G, Copeland K, Plotnick L, Kaufman F, Laffel L, Clark N;, American Diabetes Association (2005). Care of children and adolescents with type 1 diabetes: a statement of the American Diabetes Association. Diabetes Care.

[6] Azar M, Gabbay R (2009). Web-based management of diabetes through glucose uploads: Has the time come for telemedicine?. Diabetes Res Clin Pract.

[7] Kim HS (2007). A randomized controlled trial of a nurse short-message service by cellular phone for people with diabetes. Int J Nurs Stud.

[8] Tao D, Or CK (2013). Effects of self-management health information technology on glycaemic control for patients with diabetes: a meta-analysis of randomized controlled trials. J Telemed Telecare.

[9] Heidgerken AD, Lewin AB, Geffken GR, Gelfandw KM, Storch EA, Malasanos T (2005). Online diabetes education: design and evaluationwith prospective diabetes camp counsellors. J Telemed Telecare.

[10] Newton KT, Ashley A (2013). Pilot study of a web-based intervention for adolescents with type 1 diabetes. J Telemed Telecare.

[11] Whittemore R, Jaser SS, Jeon S, Liberti L, Delamater A, Murphy K, Faulkner MS, Grey M (2012). An Internet Coping Skills Training Program for Youth With Type 1 Diabetes: six-month outcomes. Nurs Res.

[12] Esmatjes E, Jansà M, Roca D, Pérez-Ferre N, del Valle L, Martínez-Hervás S, Ruiz de Adana M, Linares F, Batanero R, Vázquez F, Gomis R, de Solà- Morales O;, Telemed-Diabetes Group (2014). The Efficiency of Telemedicine to Optimize Metabolic Control in Patients with Type 1 Diabetes Mellitus: Telemed Study. Diabetes Technol Ther.

[13] Dougherty JP, Lipman TH, Hyams S, Montgomery KA (2014). Telemedicine for Adolescents With Type 1 Diabetes. West J Nurs Res.

[14] Nguyen HQ, Carrieri-Kohlman CV, Rankin SH, Slaughter R, Stulbarg MS (2004). Internet-based patient education and support interventions: A review of evaluation studies and directions for future research. Comput Biol Med.

[15] Kyngas H (2003). Patient education: perspective of adolescents with a chronic disease. J Clin Nurs.

[16] Mcmahon GT, Gomes HE, Hohne SH, Ming-Jye Hu T, Levine BA, Conlin PR (2005). Web-Based Care Management in Patients With Poorly Controlled Diabetes. Diabetes Care.

[17] Thompson DM, Kozak SE, Sheps S (1999). Insulin adjustment by a diabetes nurse educator improves glucose control in insulin-requiring diabetic patients: a randomized trial. CMAJ.

[18] Baumer JH, Hunt LP, Shield JP (1997). Audit of diabetes care by caseload. Arch Dis Child.

[19] Bin-Abbas B, Jabbari M, El-Dali A, Al-Orifi F (2014). Effect of mobile phone short text messages on glycaemic control in children with type 1 diabetes. J Telemed Telecare.

[20] Öz F (2010). Sağlık Alanında Temel Kavramlar. Hemşirelik. 2. Baskı, Ankara, Mattek Matbaacılık,.

[21] Miller EA (2007). Solving the disjuncture between research and practice: Telehealth trends in the 21st century. Health Policy.

[22] American Diabates Association (2014). Standards of medical care in diabetes-2014. Diabetes Care.

[23] Abolfotouh MA, Kamal MM, El-Bourgy MD, Mohamed SG (2001). Quality of life and glycemic control in adolescents with type 1 diabetes and the impact of an education intervention. Int J Gen Med.

[24] International Society for Pediatric and Adolescence Diabetes (ISPAD). (2011). Global IDF/Ispad Guideline For Diabetes in Childhood and Adolescence: Diabetes in Adolecent. 6th. Edition. Date accessed: 19.06.2014.

[25] Franc S, Daoudi A, Mounier S, Boucherie B, Dardari D, Laroye H, Neraud B, Requeda E, Canipel L, Charpentier G (2011). Telemedicine and diabetes: achievements and prospects. Diabetes Metab.

[26] Xu T, Pujara S, Sutton S, Rhee M (2018). Telemedicine in the Management of Type 1 Diabetes. Prev Chronic Dis..

[27] Pérez-Ferre N, Galindo M, Fernández MD, Velasco V, de la Cruz MJ, Martín P, del Valle L, Calle-Pascual AL (2010). A Telemedicine system based on Internet and short message service as a new approach in the follow-up of patients with gestational diabetes. Diabetes Res Clin Pract.

